# Use of potentially inappropriate medicines in elderly: A prospective study in medicine out-patient department of a tertiary care teaching hospital

**DOI:** 10.4103/0253-7613.64499

**Published:** 2010-04

**Authors:** H.G. Zaveri, S.M. Mansuri, V.J. Patel

**Affiliations:** Department of Pharmacology, NHL Municipal Medical College, Ahmedabad, India; 1 Department of Pharmacology, Kesar Sal Medical College, Ahmedabad, India

**Keywords:** Beers criteria, drug use study, elderly, potentially inappropriate medicines

## Abstract

**Objective:**

The present study was undertaken with the aim to detect extent of drug use in elderly at medicine outpatient department at tertiary care hospital and to evaluate inappropriate prescribing with the help of Beers' criteria 2002.

**Materials and Methods:**

The study was carried out at medicine out patient department of our hospital. 407 geriatric patients were included during the study period of three and half months. The data was collected in a proforma which included the patients' details and the prescriptions.

**Results:**

The results reveal that 7.42% of total drugs were prescribed in an inappropriate manner and 23.59% of total patients received at least one inappropriate drug prescription. Administration of a drug which is avoided in elderly forms a common category of inappropriate drug use. Antihistamines, anticholinergic, sedatives and hypnotics and cardiac glycosides are the most common drug groups prescribed in inappropriate manner.

**Conclusion:**

To conclude, this study shows high prevalence of inappropriate use of drugs in geriatric practice suggesting urgent need for sincere efforts to improve the situation.

## Introduction

The elderly population is increasing rapidly world wide.[1] About 55% of community prescriptions dispensed in 2001 in UK were meant for elderly people. However, safe and effective prescribing of medicines in elderly continues to present a major challenge.[2] In spite of the fact that elderly people are reported to be responsible for half the total drug usage, less than 5% of randomized controlled trials have been designed for people over 65 years! With limited evidence available to guide prescribing for elderly, the prescribers tend to depend on data available for younger subjects. Moreover, elderly form a heterogeneous group due to various factors like co-morbidities, interindividual variability in the aging process and interindividual differences in age-related pharmacokinetic and pharmacodynamic changes.[3] Obviously inappropriate use of drugs is expected to be high in this population.

Multiple drug use and polypharmacy is highly prevalent in elderly, exposing them not only to adverse drug reactions but also to drug interactions, increased cost of therapy, and compliance errors.[4,5] The prevalence of adverse reactions increased in the older people and reactions are reported to be more severe.[6] Studies on hospitalization due to adverse drug reactions reveal that elderly are several times more likely to be admitted due to adverse drug reactions and about half of these reactions are preventable.[6,7]

In order to prevent adverse reactions in the elderly it is important to identify the pattern of inappropriate use of medicines in this population. To evaluate the appropriateness of drugs prescribed for elderly, Beers defined criteria for potentially inappropriate medicines in 1997[8] which were updated in 2003.[9] Several studies have reported use of potentially inappropriate medicines (PIMs) in elderly people based on Beers criteria.[10‐14]

The literature related to the use of potentially inappropriate medications (PIMs) from India is scarce. Hence, this study was undertaken at a tertiary care teaching hospital with the objectives of evaluating the prevalence and pattern of PIMs using Beers criteria 2003.

## Materials and Methods

This study was carried out in medicine out-patient department (OPD) of a tertiary care teaching hospital. The hospital caters to the health care needs of millions of patients from Ahmedabad city, many villages and towns around the city and also to the patients from other neighboring states like Rajasthan, Madhya Pradesh, and Maharashtra.

The data were collected prospectively. Patients reporting to medicine OPD for treatment who were aged 65 years and above and consented for study were included. Only new cases were included in the study during the period November 2005 to February 2006. Data were collected in a proforma, which included patient's demographic details, OPD registration number, diagnosis/provisional diagnosis, and complete prescription.

### Data Analysis

About 62% drugs were prescribed by their brand names. After identification of the drugs by their generic name, they were evaluated for potentially inappropriate use with the help of Beers criteria 2003. Beers criteria are comprehensive set of explicit criteria for potentially inappropriate drug use in ambulatory elderly aged 65 years and above.[8,9]

According to these criteria, drugs which are prescribed inappropriately are classified into one of the following categories:

Category A: Drugs that generally should be avoided in older adults.

Category B: Drugs that exceed maximum recommended daily dose.

Category C: Drugs to be avoided in combination with specific co-morbidity.

### Statistical Analysis

Data obtained were analyzed with the help of SPSS software version 13. The chi-square test was used and values with *P*<0.05 were considered statistically significant.

## Results

A total 407 patients were included during the study period. Of these, 216 (53.07%) were males and 191 (46.93%) were females. The age of patients ranged from 65 years to 85 years.

### Morbidity Pattern

**Figure 1:**
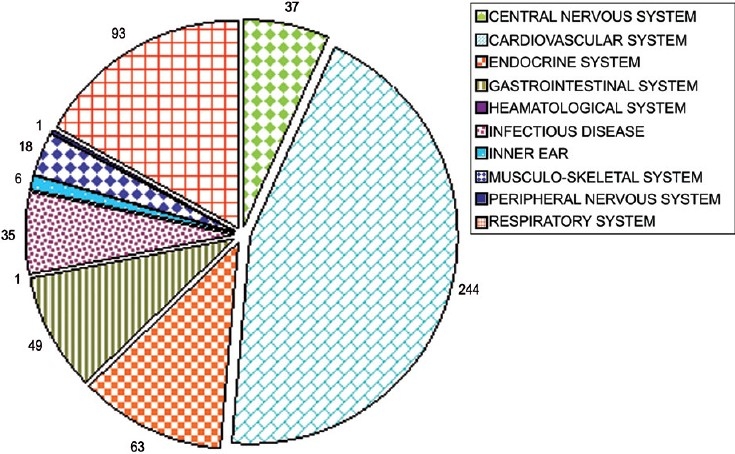
Morbidity pattern in elderly attending medicine OPD (ICD-10), n= 407

The morbidity pattern based on ICD-10[15] during the study period is shown in Figure 1. Cardiovascular disorders (59.95%) formed the most common cause for attending the OPD followed by respiratory disorders (22.85%). Hypertension (40.29%) was the most common condition affecting the geriatric patients visiting Medicine OPD, followed by diabetes mellitus (12.28%), ischemic heart disease (11.30%), upper respiratory infection (10.31%), and chronic obstructive pulmonary disease (10.31%).

### Drug Use Pattern

All 407 patients received a total 1738 drugs. The average number of drugs per patient was 4.27 (range 1 to 25). Atenolol was the most frequently used drug, being prescribed to 26.53% of patients. Other commonly prescribed drugs were paracetamol (21.86%), aspirin (19.41%), and Vitamin B complex (12.53%).

### Use of Potentially Inappropriate Medicines (Beers criteria)

Out of 407 patients, 96 patients (23.58%) received at least one drug which was potentially inappropriate and 129 out of total 1738 drugs were prescribed inappropriately; drugs to be avoided in geriatric patients (Category A) being the most common category of inappropriate use [Table 1]. According to Beers criteria 2003, out of 14 drugs prescribed inappropriately in this study, 10 carried a high degree of risk to the elderly patients. There was highly significant association between the number of drugs prescribed and frequency of use of PIMs (*P*< 0. 0002).

**Table 1:** Frequency of use of potentially inappropriate medicines in elderly*

*Category*	*Name of drugs (Severity rating)*	*Total=129*
A	Generally should be avoided in older adults	71
	Pheniramine (high)	25
	Chlorpheniramine maleate (high)	19
	Dicyclomine (high)	13
	Dextropropoxyphene (low)	10
	Amiodarone (high)	1
	Amitriptyline (high)	1
	Diazepam (high)	1
	Clonidine (high)	1
B	Drugs that exceed maximum recommended daily dose	35
	Digoxin >0.125 mg/day (low)	20
	Alprazolam >2 mg/day(high)	10
	Ferrous sulfate >325 mg/day (low)	3
	Lorazepam >3 mg/day (high)	2
C	To be avoided in combination with specific co-morbidity	23
	Phenylpropanolamine with hypertension (high)	18
	Nifedipine with constipation (low)	5

* According to Beers criteria

### Common Conditions for PIMs

Inappropriate drug use was most frequent in upper respiratory tract infection (URTI) followed by abdominal pain and congestive cardiac failure [Table 2].

**Table 2:** Common conditions for use of PIM in elderly

*Condition*	*Frequency (%)* n=*129*
Upper respiratory tract infection	51 (39.53)
Abdominal pain	23 (17.82)
Congestive heart failure	18 (13.95)
Hypertension	6 (4.65)
Insomnia	6 (4.65)

Antihitamines pheniramine and chlorpheniramine were prescribed to 6.1% and 4.6% of patients with URTI, respectively. Dicyclomine, an antimuscarinic drug, with anticholinergic properties, was used in 3.1% of elderly patients. There was no significant association between any disease condition and use of PIMs.

## Discussion

The study reveals typical morbidity pattern observed in India.[16] Cardio-vascular system (CVS) was the most common (45%) system affected. Most common indication in CVS was hypertension followed by coronary artery disease and congestive heart failure. The second most common system affected was the respiratory system, i.e. 17% of total patients. Upper respiratory tract infection was the most common respiratory condition followed by COPD.

In this study, 7.42% of total drugs prescribed were potentially inappropriate, which is higher than that reported (4.1%) by a study conducted in south India.[16] Total 96 patients out of 407, i.e. 23.59%, elderly patients received potentially inappropriate prescription of at least one drug. These findings are in agreement with a study from the Netherlands, in which 20% of ambulatory older adults received at least one potentially inappropriate drug prescription.[10] In a study carried out in Japanese long-term facility overall prevalence was 39.1%.[11] Another study in ambulatory patients reported lower prevalence at 13.4%.[19] Several studies carried out in hospitalized patients show prevalence from 25% to 49%.[12‐14,17,18]

Category A which includes drugs which should be avoided in elderly and should not be prescribed, forms a major category of inappropriate use of drugs. Beers has enlisted 46 drugs/drug groups under this category.[9] Older antihistamines (pheniramine and chlorpheniramine) prescribed to 10.7%, antispasmodic drug dicyclomine to 3.1% and dextropropoxyphene to 2.4% of patients form the majority of PIMs in category A. Other drugs like amiodarone, a class III antiarrhythmic, amitryptyline, an antidepressant drug with sedative property, clonidine, a centrally acting antihypertensive drug and diazepam, a long acting sedative hypnotic were prescribed to each patient. When compared to the Netherlands study, our figures are higher for some drugs such as antihistamines (0.1%), dextropropoxyphene (0.1%), and clonidine (0.1%), while lower use is evident for diazepam (2.8%), amitriptyline (2.0%), and amiodarone (0.7%).[10] In the Japanese study, the reported prevalence for different drugs is antihistamines (1.4%), antispasmodic (0.1%), amitriptyline (0%), and long acting benzodiazepines (0.1%).[11]

Beers criteria define maximum daily dose of certain drugs for elderly. If dose of any of these drugs exceeds the maximum dose it is considered as PIM category B, 4 drugs/drug groups being listed in this category.[9] Digoxin, a drug with narrow safety margin, was prescribed to 4.9% patients in a higher dose (>0.125 mg/day), the prevalence being higher than in the Netherlands study-0.5%[10] and 0% in the Japanese study.[11] Similarly for benzodiazepines, daily doses should not exceed 2 mg for alprazolam, 3 mg for lorazepam, 60 mg for oxazepam, and 15 mg for temazepam. In our study alprazolam was given in higher doses to 2.45% of patients and lorazepam to 0.49% of patients. This is higher compared to the Netherlands study reporting such use of alprazolam (0%) and lorazepam (0.1%) and compared to the Japanese study in which none of the patients were reported with such inappropriate use for these drugs.[10,11] According to Beers criteria if dose of ferrous sulfate exceeds 325 mg daily, then it is inappropriate. In this study 0.7% of patients received a higher dose (>325 mg/day) compared to none in the Netherlands study[10] and 0.2% in the Japanese study.[11]

Phenylpropanolamine (PPA) is still available as one of the ingredients of cough and cold preparations in India. It is inappropriate in a patient of hypertension, as it may produce elevation of blood pressure secondary to sympathomimetic activity. Hence, it is a PIM category C which includes drugs to be avoided in combination with specific co-morbidity. PPA was prescribed to 4.42% of patients with hypertension. Similarly, nifedipine, a dihydropyridine calcium channel blocker and a commonly used antihypertensive was prescribed in 1.22% of patients who had constipation. None of these two drugs was reported inappropriate by C.S. van der Hooft[10] However, Niwata *et al* reported 30.1% prevalence of use of calcium channel blockers, anticholinergics, and tricyclic antidepressants in patients with chronic constipation.[11] The same study has reported inappropriate use of NSAIDs and anti-platelet drugs in patients with clotting disorder or on anticoagulant (14.8%), use of metoclopramide and conventional antipsychotic in patients with Parkinson's disease(11.4%) and use of short/intermediate acting benzodiazepines and tricyclic antidepressants in patients with history of syncope or falls (22.3%).[11] In our study none of these PIMs which carry high risk according to Beers criteria were observed, which is a positive finding.

Studies to identify the factors for PIM have reported older patients, polypharmacy, depression immobilization, and hypertension as some of the factors associated with increased risk of PIM.[12,19] In our study polypharmacy is the only factor associated with use of PIMs, while disease condition and age did not show significant association.

Some other criteria also exist for evaluating the use of PIMs in elderly. In a study designed to compare PIM prevalence rates based on the 1997 Beers criteria and Zhan criteria with the rate obtained using the 2003 Beers criteria, the prevalence was estimated at 13.4% based on the 2003 Beers criteria, compared with 8.8% based on the 1997 Beers criteria and 4.2% based on the Zhan criteria.[20] STOPP (Screening Tool of Older Persons' potentially inappropriate Prescriptions) is a new, systems-defined medicine review tool. In a study comparing the performance of STOPP to that of established Beers criteria in detecting potentially inappropriate medicines (PIMs) and related adverse drug events in older patients presenting for hospital admission, STOPP criteria identified a significantly higher proportion of patients requiring hospitalization as a result of PIM-related adverse events than Beers criteria.[21]

## Conclusion

This study suggests that use of PIMs is common in elderly patients, some of them associated with high degree of risk in terms of adverse drug reactions or worsening of the co-morbidity. Evidence indicates that high prevalence of inappropriate prescribing of medicines in elderly people is associated with increased morbidity and mortality, increased cost, and decreased quality of life. Our study has been limited to only one specialty. More studies in other specialties and general practice are necessary to sensitize the practitioners to this important public health issue.
